# SUPR-3D: A randomized phase iii trial comparing simple unplanned palliative radiotherapy versus 3d conformal radiotherapy for patients with bone metastases: study protocol

**DOI:** 10.1186/s12885-019-6259-z

**Published:** 2019-10-28

**Authors:** Robert Olson, Roel Schlijper, Nick Chng, Quinn Matthews, Marco Arimare, Lindsay Mathews, Fred Hsu, Tanya Berrang, Alexander Louie, Benjamin Mou, Boris Valev, Joanna Laba, David Palma, Devin Schellenberg, Shilo Lefresne

**Affiliations:** 1 University of British Columbia, Vancouver, Canada; 2 University of Northern British Columbia, Prince George, Canada; 3 Department of Radiation Oncology, BC Cancer, 1215 Lethbridge Street, Prince George, BC V2M7A9 Canada; 4 BC Cancer, Abbotsford, Canada; 5 BC Cancer, Victoria, Canada; 6 Sunnybrook Health Sciences Centre, Toronto, Ontario Canada; 7 BC Cancer, Kelowna, Canada; 8 London Health Sciences Centre, London, Ontario Canada; 9 BC Cancer Surrey, Surrey, British Columbia Canada; 10 BC Cancer, Vancouver, Canada

**Keywords:** Bone metastases, Radiotherapy, Quality of life, Radiation-induced nausea and vomiting

## Abstract

**Background:**

Bone metastases in the lower spine and pelvis are effectively palliated with radiotherapy (RT), though this can come with side effects such as radiation induced nausea and vomiting (RINV). We hypothesize that high rates of RINV occur in part because of the widespread use of inexpensive simple unplanned palliative radiotherapy (SUPR), over more complex and resource intensive 3D conformal RT, such as volumetric modulated arc therapy (VMAT).

**Methods:**

This is a randomized, multi-centre phase III trial of SUPR versus VMAT. We will accrue 250 patients to assess the difference in patient-reported RINV. This study is powered to detect a difference in quality of life between patients treated with VMAT vs. SUPR.

**Discussion:**

This trial will determine if VMAT reduces early toxicity compared to SUPR and may provide justification for this more resource-intensive and costly form of RT.

**Trial registration:**

Clinicaltrials.gov identifier: NCT03694015.

Date of registration: October 3, 2018.

## Background

Bone metastases are the most common site of distant metastases in oncologic patients. There is a high incidence of bone metastases in the pelvis and lower spine, often causing pain which can significantly impact a patient’s quality of life [1]. Palliative radiotherapy (RT) is an effective treatment for bone metastases, resulting in significant pain reduction in the majority of patients [2]. It is also effective in preserving function and maintaining skeletal integrity, while minimizing the occurrence of adverse skeletal related events [3]. In many centres, bone metastases are treated using a Simple Unplanned Palliative Radiation (SUPR) technique using static fields. This technique requires minimal contouring and dosimetric calculations, and less stringent dosimetric quality review, making this a time- and cost-effective treatment technique.

SUPR is associated with irradiation of normal tissue within the treatment field since the entire portal is exposed to the prescribed dose. While fatigue, pain flare, and erythema in the irradiated area are relatively common adverse effects associated with treating bone metastases, site-specific toxicity can also occur, including esophagitis, nausea, or diarrhea when dose is delivered to the gastro-intestinal tract. The majority of patients treated with SUPR to the pelvis and lower spine suffer from radiation induced nausea and vomiting (RINV) due to incidental bowel irradiation [4]. This potentially greatly affects quality of life in these patients, for whom quality of life is the cornerstone of treatment. By using more complex 3D conformal RT like volumetric modulated arc therapy (VMAT), the dose to the intestines can be decreased whilst still treating the bone metastases to an effective dose, possibly reducing early and late toxicity after palliative RT. [5–7]

**Fig. 1:**
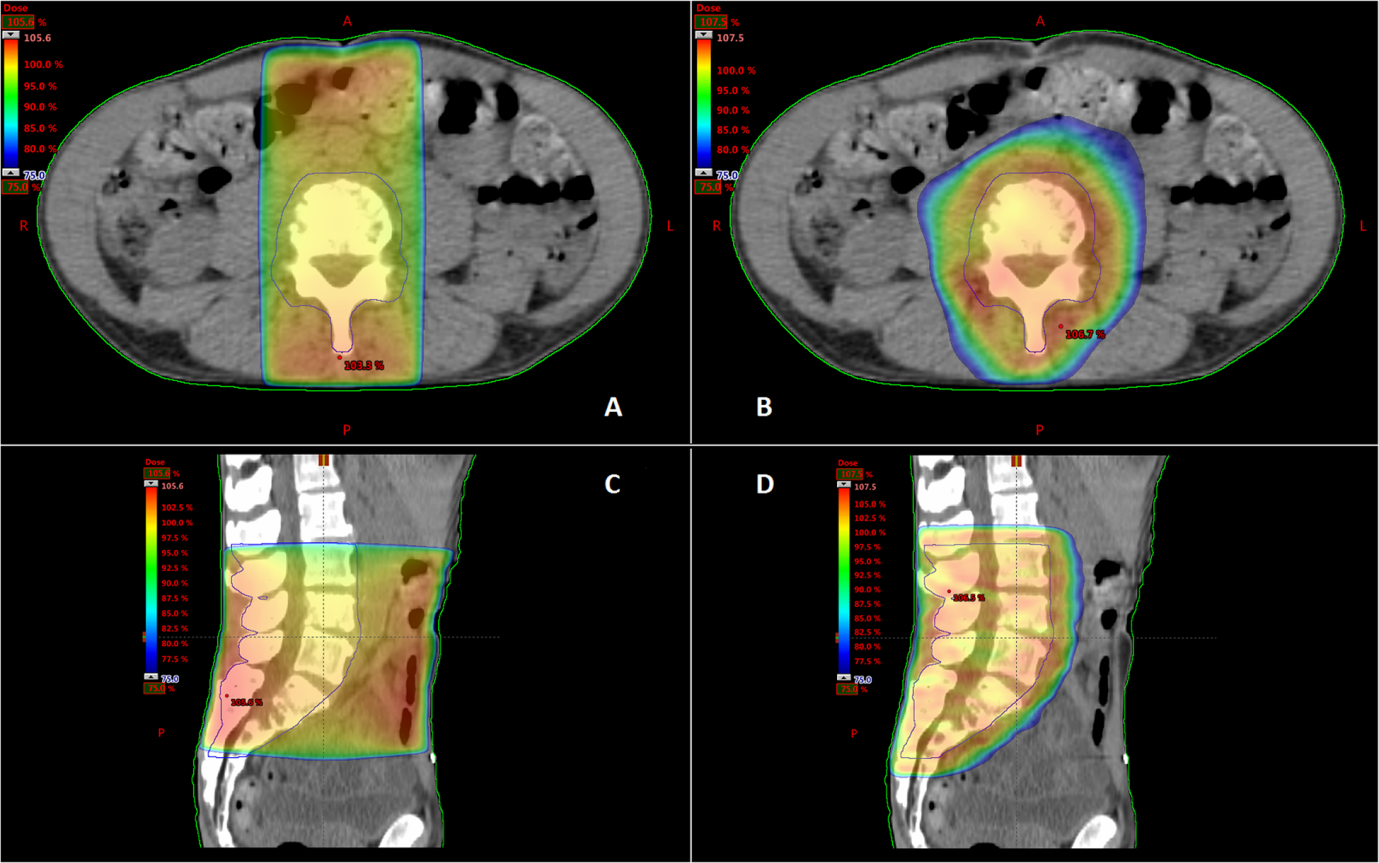
Dose distributions for a SUPR plan (**a**, **c**) and VMAT plan (**b**, **d**)

In SUPR, radiation dose is delivered using one or two static radiation fields with a fixed shape. In contrast, VMAT delivers the radiation dose in a continuous rotation of the radiation source, allowing treatment from a 360° beam angle with continuous modulation of the beam shape and intensity. This results in a highly conformal dose distribution with improved target coverage, while better sparing normal tissue [5] (Fig. 1). However, VMAT includes more complex planning and quality assurance (QA) processes compared to SUPR [8]. This can be expensive and time-consuming, which can have a significant impact on departmental resources and wait time for patients [9]. Therefore, it is important to demonstrate that VMAT results in a reduction of RINV to justify increased costs and longer waiting times for patients.

The current standard of care in many Canadian and European centres for palliative patients with bone metastases is SUPR. To the best of our knowledge, there is no level I evidence supporting the use of VMAT for palliative patients with bone metastases. The goal of this study is to investigate whether the use of VMAT in these patients is warranted. We hypothesize that VMAT will reduce RINV in palliative patients treated for bone metastases in the lower spine and pelvic regions as compared to patients treated with SUPR.

## Methods/design

This is a randomized, multi-centre phase III trial where 250 participants will be randomized between SUPR or 3D conformal palliative RT using VMAT. The study has been approved by the University of British Columbia Research Ethics Board in compliance with the Helsinki Declaration.

### Objectives

The primary objective is to compare patient-reported Quality of Life related to RINV between standard palliative radiotherapy and VMAT. Secondarily, we will assess rate of complete control of RINV, compare patient reported toxicity, and evaluate pain response. However, we hypothesize that there will be no difference in pain response between the two arms, because they are receiving the same dose.

#### Primary endpoint

- Patient reported Quality of Life related to Radiation Induced Nausea and Vomiting (RINV) as scored by the Functional Living Index – Emesis (FLIE) at day 5 post RT start.

#### Secondary endpoints

- Primary efficacy outcome.
  - Control of RINV measured by a daily patient diary (day 1–5)
- Secondary Patient Reported Outcomes (see Additional file 1).
  - Pain flare measured by the Brief Pain Inventory (BPI)
  - Diary of medication use (specifically anti-emetics)
  - Fatigue (PRO-CTCAE)
  - PRO-CTCAE:
    - Decreased appetite
    - Nausea
    - Vomiting
    - Diarrhea
    - Radiation skin reaction
    - Pain flare
    - Fatigue

- Pain response assessed by the Brief Pain Inventory.
- Proportion of patients who receive treatment within 1 day, 2 days, 3 days, 4 days, 5 days or more than 5 days.
- Toxicity assessed by HCP (Healthcare Professional) reported baseline and follow-up (Medications and Toxicity).
- Quality of Life: single item from EORTC QLQ C-15 PAL: ‘How would you rate your overall quality of life during the past week’.

### Study design

This study is a multicentre randomized trial. Participating centres will be tertiary, academic hospitals or radiotherapy treatment centres in Canada. Patients will be randomized in a 1:1 ratio between Arm 1 or Arm 2 with stratification for prescribed dose.

### Entry procedures

All randomizations will be done using a computer-generated randomization scheme.

All eligible patients enrolled in the study by the participating treatment centre will be assigned a study number, which must be used on all documentation.

The following information will be required

- Trial Code
- Name of investigator under whose name the patient will be randomized
- Informed consent, version date, date signed by patient, name of person conducting consent discussion and date signed by the person who conducted the consent form discussion
- Confirmation that the patient meets the eligibility requirements
- Stratification factors

### Randomization

**Fig. 2:**
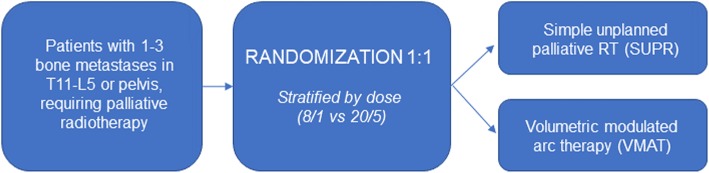
Study design

Simple randomization with stratification for 8 Gray (Gy) single fraction vs. 20 Gy in 5 fractions will be used to randomly assign patients to either Arm 1 or Arm 2 in a 1:1 ratio (Fig. 2) using a computer-generated randomization scheme. Randomization will be performed on patient-level, meaning that if a patient is treated for multiple bone metastases in the same course, all will receive the same treatment technique. The randomization sequence is known only to the statistician and uploaded into a restricted-access database (REDCap) housed on secure hospital servers at BC Cancer. Upon enrollment of a patient, the database will be accessed by the trial coordinator to obtain the next intervention in the random sequence, which will then be assigned to the patient.

### Intervention

Patients randomized to the intervention group will be treated with palliative radiotherapy using a VMAT technique.

### Inclusion criteria

- Age 18 or older
- Able to provide informed consent
- Clinical diagnosis of cancer with bone metastases (biopsy of treated bone metastases not required)
- Currently being managed with palliative intent RT to 1–3 bone metastases, at least one of which must (at least) partly lie within T11-L5 or pelvis.
- Eastern Cooperative Oncology Group (ECOG) Performance Status 0–3
- Patient has been determined to potentially benefit from 8 Gy or 20 Gy
- Radiation Oncologist (RO) is comfortable prescribing 8 Gy in 1 fraction or 20 Gy in 5 fractions RT for bone metastases
- Pregnancy test for women of child-bearing potential
- Patient is able (i.e. sufficiently fluent) and willing to complete the patient-reported outcomes questionnaires in English. The baseline assessment must be completed within required timelines, prior to randomization.
- Patients must be accessible for treatment and follow-up. Investigators must assure themselves the patients randomized on this trial will be available for complete documentation of the treatment, adverse events, and follow-up.
- For simplicity of planning, expected Gross Tumor Volume (GTV) should be less than 20 cm based on radiological or clinical evidence.
- Patient must be prescribed a 5HT-3 receptor antagonist (e.g. Ondansetron) as antiemetic prophylaxis prior to RT start.

### Exclusion criteria

- Serious medical co-morbidities precluding RT
- Clinical evidence of spinal cord compression
- Spinal cord in treatment field has already received at least 30 Gy EQD2
- Whole brain radiotherapy within 4 weeks of RT start
- Systemic therapy during and 1 week prior/after radiation
- Solitary plasmocytoma
- Pregnant or lactating women
- Target volume cannot be encompassed by a single VMAT isocentre
- Custom mould room requirements (shells and other immobilization that is standard-of-care is acceptable)
- Greater than two organs-at-risk requiring sparing during VMAT optimization.
- Patients requiring treatments outside standard clinical hours
- Implanted electronic device within 10 cm of the RT fields
- Prostheses in the axial plane of the target, or within 1 cm of the Planning Target Volume (PTV) out-of-plane
- Previous RT that requires an analysis of cumulative dose (i.e. sum plans or EQD2 calculations)
- Oral or IV contrast if the local standard-of-care requires compensation for this in planning.

### Pre-treatment assessment (baseline)

- ECOG status
- Eligibility according to inclusion- and exclusion criteria
- Patient reported outcomes
  - Brief Pain Inventory

### Radiation technique

- All metastases lying at least partly within T11-L5 or the pelvis will be treated according to randomization technique.
- All treated metastases included in this trial should receive the same dose, chosen pre-randomization.
- Radiation doses allowed are either 8 Gy in 1 fraction or 20 Gy in 5 fractions.
- All other bone metastases that need to be treated can be treated at the same time with either 8 Gy in 1 fraction or 20 Gy in 5 fractions. Technique for these lesions can be chosen by RO or centre discretion.
- The total number of fields that can be treated synchronously is 3, including both eligible and ineligible fields.
- If additional bone metastases are symptomatic, they can be treated at a later time, no sooner than 4 weeks from the end of RT on trial.

### Radiation treatment planning for SUPR

#### Planning according to local protocols

No more than 2 fields; no beam modifying devices, other than multileaf collimators (MLCs). Alternate weighting of beams allowed (i.e. 1:2 anterior-posterior). Review of dosimetry not required, if performed as per institutional standard.

Minimum of kiloVolt image matching on unit daily.

### Radiation treatment planning for VMAT

#### Contouring

GTV: based on available imaging (GTV may be based on Computed Tomography (CT) simulation scan alone; no special imaging is required) and is expected to be between 1.5 cm and 20 cm clinically.

Clinical Target Volume (CTV) = GTV + 0.5 to 0.7 cm (RO preference), adjusted to the anatomy.

- In case of only bone involvement: no margin outside the bone
- In case of bone and soft tissue involvement: no margin outside the bone, only adapt CTV margin in soft tissue to organs. No CTV adaptation in i.e. muscle.
- CTV may be optional and if used can encompasses whole vertebral body as per RO’s discretion

(Note: CTV is optional if confident in GTV and PTV)

PTV = CTV (or GTV) + (1 to 1.5) cm as per RO / centre preference.

PTV_eval = PTV cropped 0.5 cm below skin.

Organs at Risk (OAR’s): A maximum of 2 OAR’s are permitted for the VMAT arm. OAR contouring and constraints are at the discretion of the treating RO. However, if lung/kidneys are within 5 cm of the PTV, the absence of constraints for these contours should be documented in the treatment plans or dose constraint sheet prior to planning. PTV can be compromised for OAR at radiation oncologist’s discretion. Kidneys are considered 1 organ.

### Planning

- AAA or other type-2 / model-based calculation framework
- Heterogeneity corrections applied
- Maximum calculation grid size = 2.5 mm
- Planning VMAT flash is permitted but not required
- Jaw-tracking is permitted but not required
- A normal tissue constraint should be used to control conformity to at least the 65% isodose level
- For the VMAT arm, up to two arcs are permitted

#### Required constraints

- PTV / PTV_eval coverage: The volume of the PTV covered by the 95% isodose volume must be greater than or equal to 98% (V95% ≥ 98%) (V95% < 98% minor violation; V95% < 50% major violation)
- The 80% conformity index (CI) must be less than 1.75 (1.75–1.9 major violation)
- Plan maximum dose (Dmax) = 110% (> 110% but ≤115% minor violation; > 115% major violation)
- Maximum of 2 constrained OAR’s
- In case of accomplished constraints for CI, Dmax and OAR’s (if present): no further plan modification permitted by RO

#### Suggested constraints

Recommended OAR constraints are given in Table 1 below, which are based on QUANTEC, adapted to the specific dose per fraction of the two schedules using EQd2. The decision to include or adjust these constraints is at the discretion of the RO.

**Table 1:** Suggested constraints

	8 Gy in 1 fraction	20 Gy in 5 fractions
^a^Spinal Cord	Max dose < 110% of 8 Gy	Max dose < 110% of 20 Gy
Lungs (excl. GTV)	V6 Gy < 35%	V12 Gy < 35%
	Mean dose < 6 Gy	Mean dose < 12 Gy
Kidney (each)	V6 Gy < 30%	V12 < 30%
	Mean dose < 5 Gy	Mean dose < 10 Gy
^b^Small Bowel	Max dose < 110% of 8 Gy	Max dose < 110% of 20 Gy

^a^ spinal cord to L2, spinal cord PRV is 0.5 cm margin around the spinal cord

^b^ small bowel contoured by RO or RT depending on institutional polices

#### Plan review and QA

No pre-treatment dosimetric review is required if both the required and RO-specified OAR constraints are met. Otherwise, the plan must be reviewed by the RO prior to treatment. Document any further plan modification secondary to subsequent local QA procedures as a minor protocol violation. Physics and dosimetry checks are to be performed as per local standard-of-care.

#### Verification imaging

(Image Guided Radiotherapy (IGRT): Minimum IGRT is daily 2D kV matching. Cone-beam CT (CBCT) is not required but may be used at the discretion of the treating radiation oncologist.

#### Nausea prophylaxis

All patients will receive a 5HT-3 receptor antagonist (e.g. Ondansetron) as anti-emetic prophylaxis prior to RT start. Dexamethasone may also be given for nausea prevention, though is not mandated.

### Quality assurance

Dosimetric compliance with protocol constraints will be evaluated by the planning dosimetrist(s). Plan review by the radiation oncologist is not required for both arms. The radiation oncologist might review the plan but no plan modification at that point is permitted.

For VMAT, patient-specific QA should be performed per standard processes. Institutional QA rounds may also evaluate the radiation plans.

### Data safety monitoring committee

There is no independent data safety monitoring committee (DSMC) for this study. The DMSC will be made up of the study co-investigators. The DSMC will meet twice annually after study initiation to review toxicity outcomes. If any grade 3–5 toxicity is reported, the DSMC will review the case notes to determine if such toxicity is related to treatment. If the DSMC deems that toxicity rates are excessive (> 25% grade 3 toxicity, or > 10% grade 4 or > 3% grade 5 toxicity), then the DSMC can, at its discretion, recommend cessation of the trial, dose adjustment, or exclusion of certain treatment sites that are deemed as high-risk for complications.

### Subject discontinuation/withdrawal

Subjects may voluntarily discontinue participation in the study at any time. If a subject is removed from the study, the clinical and laboratory evaluations that would have been performed at the end of the study should be obtained. If a subject is removed because of an adverse event, they should remain under medical observation as long as deemed appropriate by the treating physician.

### Follow-up schedule

See Table 2 for follow-up schedule.

**Table 2:** Follow-up schedule

	Pre-Treatment		Treatment		Follow-Up		Early Termination
Tests & Procedures	Recruitment	Enrollment/ Baseline	Day 1	Day 5	Week 2 Day 14 (+/− 3 days)	Week 4 Day 28 (+/− 3 days)	(collect only if patient allows/agrees)
Pre-Screen	X						
Informed Consent	X						
Eligibility Screen		X					
^a^History and physical exam		X					
^a^Pregnancy Test (if applicable)		X					
Patient Diary (provided to patient)			X	X Day 1–5			
^b^ Brief Pain Inventory		X	X		X	X	X
Functional Living Index - Emesis		X	X		X	X	X
PRO-CTCAE & QoL EORTC QLQ-C15-PAL			X		X	X	X
Treatment Related Data			X				
HCP-reported baseline and follow up form (Medications and Toxicity)		X			X	X	X

^a^ *may be done within 90 days, or 3 months, prior to enrollment*

^b^ *BPI on Day 1 does not need to be administered again if collection was done within 1 week of baseline*

### Physician/registered nurse (RN)/other reported outcomes

- HCP-reported baseline and follow-up
  - Outcome
    - ECOG status
    - Medication use
  - Toxicity (CTCAE v5.0)
    - Pain
    - Fatigue
    - Diarrhea
    - Nausea

### Treatment response evaluation

#### FLIE

Scores on all individual questions will be weighted equally, reversed if required and summed to create an overall FLIE score between 18 and 126. Scores will then be normalized with a range from 0 to 108 for ease of interpretation on figures in the manuscript. A low score is favorable, reflecting less nausea and vomiting.

#### RINV

Complete control: no increased episodes of nausea or vomiting with no increased use of anti-emetic medication from baseline.

Partial control: 1–2 increased episodes of nausea or vomiting with no increased use of anti-emetic medication from baseline.

Uncontrolled response: 3 or more increased episodes of nausea or vomiting, or increased use of anti-emetic medication from baseline.

Overall control: includes complete and partial control.

#### Pain

*Complete response:* pain score of 0 at treated site with no increase in analgesic intake (stable or reducing analgesics in daily oral morphine equivalent dose (OMED).

Partial response: pain reduction of 2 or more at the treated site on a scale of 0 to 10 without analgesic increase, or analgesic reduction of 25% or more from baseline without an increase in pain.

Pain progression: Increase in pain score of 2 or more above baseline at the treated site with stable OMED, or an increase of 25% or more in OMED from baseline with the pain score stable or 1 point above baseline.

Indeterminate response: Any response that is not captured by the complete response, partial response or pain progression definitions [10].

### Statistical analysis

#### Sample size

The primary outcome is Functional Living Index – Emesis (FLIE) score compared between the two arms at day 5 post start of RT. Based on previous literature, we expect both arms to have a relatively normal (i.e. score of 0) FLIE score at baselines. We expect patients in the SUPR arm to have a mean FLIE score of 18, 5 days post start of RT. [11] We anticipate that VMAT will have a much lower RINV impact (i.e. less decline in FLIE) and for the purpose of this study will hypothesize that the FLIE will be approximately 10.

Sample size was calculated with these FLIE scores. With alpha Type I error set at 0.05 and power set at 0.9, with a dropout rate of 30%, we calculated a conservative sample size of 250 patients.

Our most important secondary outcome (primary efficacy outcome) is RINV which occurs in 60% of patients who receive RT to the lower spine and pelvis [4, 11]. Using the sample size of 250 patients (see above), this study has a power of 0.8 to detect a 25% difference in RINV (from 60 to 35%, see Table 3.) with alpha Type I error set at 0.05 and a dropout rate of 20%. As outlined in the table below, if RINV difference is lower or higher, our power will be lower and higher, respectively.

**Table 3:** Sample sizes to detect differences in RINV

	Approximate sample size required
RINV 60 to 50%	1600
RINV 60 to 40%	400
RINV 60 to 35%	250
RINV 60 to 30%	175
RINV 60 to 20%	90

#### Analysis plan

Patients will be analyzed in the groups to which they are assigned (intention-to-treat). De-identified data (except for study number and initials, see confidentiality below) will be transmitted from participating centres via REDCap to be collected centrally where it will be stored on secure hospital servers at BC Cancer. Source documents will also be uploaded. Research coordinators (clinical trials staff) will perform data checks throughout the trial period and will call participating centres or visit as necessary. Patients in both arms will receive the same radiation dose. Therefore we do not expect a difference in toxicity or other safety concerns. Thus, we will not conduct an interim-analysis and there will be no stopping rules. All outcomes based on means will be analysed using the students t-test. All proportions will be analysed using chi-square test.

#### Confidentiality

The names and personal information of study participants will be held in strict confidence. All study records (case report forms, safety reports, correspondence, etc.) will only identify the subject by initials and the assigned study identification number. The investigator will maintain a confidential subject identification list (Master List) during the course of the study. Access to confidential information (i.e., source documents and patient records) is only permitted for direct subject management and for those involved in monitoring the conduct of the study (i.e., Sponsors, CRO’s, representatives of the IRB/REB, and regulatory agencies). The subject’s name will not be used in any public report of the study.

#### Data sharing statement

Deidentified participant data from this trial will not be shared publicly, however, the full protocol will be published along with the primary analysis of the outcomes.

#### Protocol amendments and trial publication

Any modifications to the trial protocol must be approved and enacted by the principal investigator. Protocol amendments will communicated to all participating centres, investigators, IRBs, and trial registries by the principal investigator. Any communication or publication of trial results will be led by the principal investigator, and is expected to occur within 1 year of the primary analysis. Trial results will remain embargoed until conference presentation of an abstract or until information release is authorized. Authorship of the trial abstract and ultimately the full manuscript will be decided by the principal investigator at the time of submission. Professional writers will not be used for either abstract or manuscript preparation.

## Discussion

This study has been designed to compare early toxicity between two radiation treatment techniques currently used for palliative treatment of bone metastases, with vastly different resources required to implement. The primary potential advantage of VMAT over SUPR is the conformality of radiation dose to the target, and avoidance of normal tissue, such as bowel. Theoretically, this should lead to less RINV in the population eligible for this trial, though we believe this should be assessed in randomized trials before widespread adoption of this expensive and resource intensive technique is more widely adopted. Many radiation centres world-wide have already implemented the use of more advanced radiation techniques like VMAT for palliative patients. This trial has the potential of proving no difference between SUPR and VMAT which might lead to the need for revisions of local treatment protocols. If the outcome in both arms is equal, centres might want to decrease the use of VMAT for palliative patients with advantages regarding planning time and costs. However, even if the outcomes in this trial are similar for both arms, VMAT might still be warranted in certain scenarios. There are many reasons to choose one technique over the other. The decision on which treatment technique will be used has to be made on an individual patient level, where possible in a shared decision-making setting. We hypothesize that with this trial, we are able to provide evidence that can improve this decision-making process.

## Supplementary information

**Additional file 1: Appendix 1.** Eligibility criteria. **Appendix 2.** Patient reported outcomes. **Appendix 3:** HCP-reported baseline and follow-up. **Appendix 4.** Treatment related data. **Appendix 5**. Informed consent form.

## Data Availability

Not applicable.

## Abbreviations

CBCT: Cone-beam CT
CT: Computed Tomography
CTV: Clinical Target Volume
ECOG: Eastern Cooperative Oncology Group
GTV: Gross Tumor Volume
Gy: Gray
IGRT: Image guided radiotherapy
OAR’s: Organs at Risk
PTV: Planning Target Volume
BPI: Brief pain inventory
CI: Conformity index
DSMC: Data safety monitoring committee
FLIE: Functional living index – emesis
HCP: Health care professional
MLC: Multileaf collimator
OMED: Oral morphine equivalent dose
QA: Quality assurance
RINV: Radiation-induced nausea and vomiting
RN: Registered nurse
RO: Radiation oncologist
RT: Radiotherapy
SUPR: Simple unplanned palliative radiotherapy
VMAT: Volumetric modulated arc therapy
