# Supplementary material for: SUPR-3D: A randomized phase iii trial comparing simple unplanned palliative radiotherapy versus 3d conformal radiotherapy for patients with bone metastases: study protocol
Source: BMC Cancer. 2019 Oct 28;19:1011. doi: 10.1186/s12885-019-6259-z (PMC6819327; doi:10.1186/s12885-019-6259-z)
Supplement: Supplementary file 1 — Additional file 1: Appendix 1. Eligibility criteria. Appendix 2. Patient reported outcomes. Appendix 3: HCP-reported baseline and follow-up. Appendix 4. Treatment related data. Appendix 5. Informed consent form. [file 12885_2019_6259_MOESM1_ESM.zip › APPENDIX AR3.docx]

# APPENDIX A – ELIGIBILITY CRITERIA

**Eligibility Checklist**

You must be able to circle **Y (Yes)** to all of the following:

Y / N: Age 18 or older

Y / N: Able to provide informed consent

Y / N: Clinical Diagnosis of cancer with bone metastases

Y / N: Currently being managed with palliative intent RT to 1-3 bone metastases, at least one of which must (at least partly) lie within T11-L5 or pelvis

Y / N: ECOG Performance Status 0-3

Y / N: Radiation Oncologist is comfortable prescribing 8 Gy in 1 fraction or 20 Gy in 5 fractions RT for bone metastases

Y / N: Patient has been determined to potentially benefit from 8 Gy or 20 Gy

Y / N / NA: Pregnancy test for women of child-bearing potential

Y / N: Patient is able (i.e. sufficiently fluent) and willing to complete the quality of life questionnaire

Y / N: Patients must be accessible for treatment and follow-up

Y / N: Radiological or clinical evidence confirming GTV is expected to be less than 20 cm

Y / N: Patient will be/has been prescribed a 5HT-3 receptor antagonist (e.g. Ondansetron) as antiemetic prophylaxis prior to RT start.

**Exclusion Criteria**

You must be able to circle **N (No)** to all of the following:

Y / N: Serious medical co-morbidities precluding radiotherapy

Y / N: Clinical or radiological evidence of spinal cord compression

Y / N: Systemic therapy during and one week prior/after radiation

Y / N: Solitary plasmacytoma

Y / N: Pregnant or lactating women

Y / N: Target volume cannot be encompassed by a single VMAT isocentre

Y / N: Custom mould room requirements (shells and other immobilization that is standard-of-care is acceptable)

Y / N: Greater than two organs-at-risk requiring optimization.

Y / N: Spinal cord in treatment field has already received at least >30 Gy EQD2

Y / N: Implanted electronic device within 10 cm of the RT fields

Y / N: Prostheses in the axial plane of the target, or within 1 cm of the PTV out-of-plane

Y / N: Previous RT that requires an analysis of cumulative dose (i.e. sum plans or EQD2 calculations)

Y / N: Oral or IV contrast if the local standard-of-care requires compensation for this in planning.

Y / N: Patient requires treatment outside standard clinical hours
