# Supplementary material for: SUPR-3D: A randomized phase iii trial comparing simple unplanned palliative radiotherapy versus 3d conformal radiotherapy for patients with bone metastases: study protocol
Source: BMC Cancer. 2019 Oct 28;19:1011. doi: 10.1186/s12885-019-6259-z (PMC6819327; doi:10.1186/s12885-019-6259-z)
Supplement: Supplementary file 1 — Additional file 1: Appendix 1. Eligibility criteria. Appendix 2. Patient reported outcomes. Appendix 3: HCP-reported baseline and follow-up. Appendix 4. Treatment related data. Appendix 5. Informed consent form. [file 12885_2019_6259_MOESM1_ESM.zip › APPENDIX BR3.docx]

**APPENDIX B – PATIENT-REPORTED OUTCOMES**

All patients:

- Daily diary (day 1-5) completed by patient (see next page)
- Brief Pain Inventory (POSI) Questionnaire (paper or iPAD) completed by patient
- FLIE completed by patient
- PRO CTCAE & QoL EORTC QLQ-C15-PAL
  - Fatigue measured with PRO CTCAE completed by patient
    - In the last 7 days, what was the severity of your fatigue, tiredness, or lack of energy at its worst?
    - In the last 7 days, how much did fatigue, tiredness, or lack of energy interfere with your usual or daily activities?
  - Skin: PRO-CTCAE
    - In the last 7 days, what was the severity of your skin burns from radiation at their worst?
  - QoL EORTC QLQ-C15-PAL (version 1) Question 15:
    - How would you rate your overall quality of life during the past week?

**Patient Diary**

**Date of Tx**: _____-_____-_____

yyyy-mmm-dd

**Day**: ___ of **___**

.

Please **CIRCLE** whether or not you have had any of the following side effects in the last 24 hours and rate its severity (mild, moderate or severe).

| SYMPTOM | SEVERITY | | | |
| --- | --- | --- | --- | --- |
| **1. Nausea (feeling sick to your stomach)** | **NONE** | **MILD** | **MODERATE** | **SEVERE** |
| **2. Vomiting / Retching (being sick to your stomach)** | **NONE** | **MILD** | **MODERATE** | **SEVERE** |
| **3. Diarrhea** | **NONE** | **MILD** | **MODERATE** | **SEVERE** |

.

In the past 24 hours, how many times have you felt **nauseated** (i.e. feeling sick to your stomach)?

0 1 2 3 4 5 6 7 or more (specify:____)

.

In the past 24 hours, how many times have you **vomited/retched** (i.e. being sick to your stomach)?

0 1 2 3 4 5 6 7 or more (specify:____)

Please record all **regular/prophylactic** nausea and vomiting medications you had taken during the past 24 hrs (i.e. pills taken prior to actual onset of symptoms)

| Name of medication | # of pills taken at once | Strength (mg/pill) | # of pills in past 24 hours |
| --- | --- | --- | --- |
|  |  |  |  |
|  |  |  |  |
|  |  |  |  |

Please record all **break-through** nausea and vomiting medications you had taken during the past 24 hrs

(i.e. pills taken once you started to feel sick).

| Name of medication | # of pills taken at once | Strength (mg/pill) | # of pills in past 24 hours |
| --- | --- | --- | --- |
|  |  |  |  |
|  |  |  |  |
|  |  |  |  |

How much **relief** did your medications provide from nausea and/or vomiting? **□ N/A**

| **0%** | 10% | 20% | 30% | 40% | 50% | 60% | 70% | 80% | 90% | **100%** |
| --- | --- | --- | --- | --- | --- | --- | --- | --- | --- | --- |

**No relief**  **Complete relief**

Did you feel nauseated and/or did you vomit at any time during the past 24 hours? **□ NO □ YES**

If you answered **YES**, please answer the **5** questions below.

| **During the past 24 hours, did your**  **nausea or vomiting:** | **Not at All** | **A**  **Little** | **Quite a Bit** | **Very Much** |
| --- | --- | --- | --- | --- |
| 1. Interfere with your appetite? | 1 | 2 | 3 | 4 |
| 1. Affect your sleep? | 1 | 2 | 3 | 4 |
| 1. Interfere with your physical activities (such as looking after yourself or going outdoors)? | 1 | 2 | 3 | 4 |
| 1. Interfere with your social life? | 1 | 2 | 3 | 4 |
| 1. Interfere with your enjoyment of life?   **Report completed by:** □ Participant without assistance  □ Participant with assistance from family/friend  □ Participant with assistance from Research Coordinator | 1 | 2 | 3 | 4 |

**Brief Pain Inventory (attachment)**


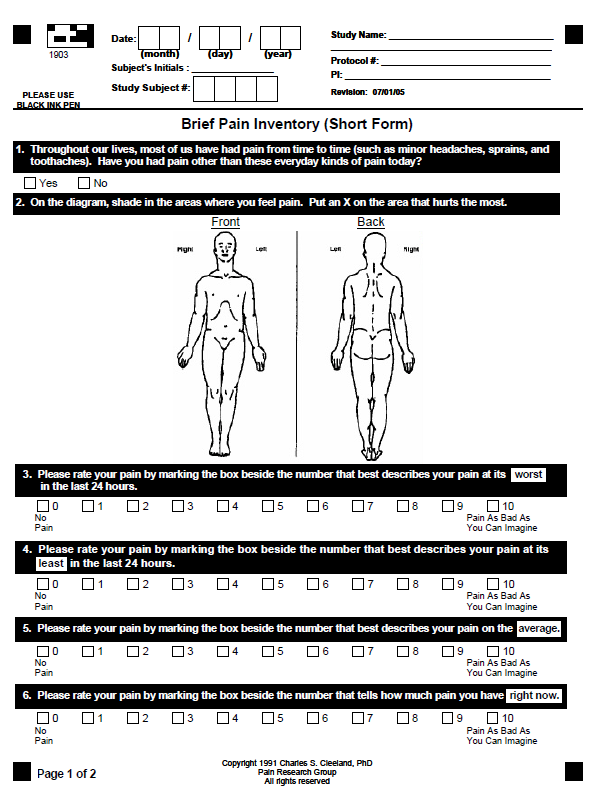


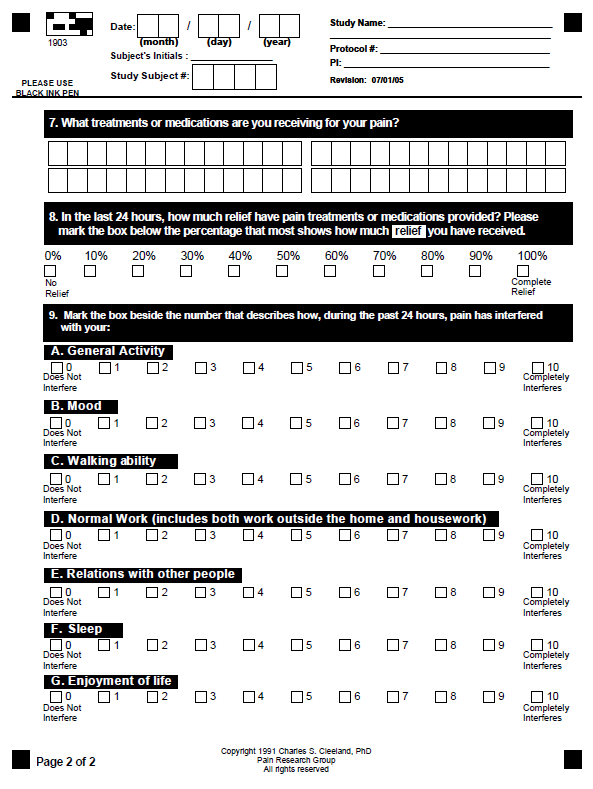


Study ID________

| **Functional Living Index - Emesis**  **PATIENT INSTRUCTIONS** | |
| --- | --- |
| In the following questionnaire you are asked to rate to what extent nausea and vomiting have affected your quality of life. The first set of 9 questions refers to nausea and the second set of 9 questions refers to vomiting. The questionnaire should take approximately 10 minutes or less to complete. Please read the instructions before you begin. Think carefully about each question because your answers may help to develop treatments that will improve the quality of life for future patients. | |
| For each question, you will rate to what extent nausea (or vomiting) has affected an aspect of your quality of life during the past five days. Please focus on your experiences **over that time period**. We are interested in **your opinions**, not those of family members or friends. Your answers will remain confidential. | |
| You must answer every question. Use a black ballpoint pen and press firmly so that your mark is clear. | |
| If you are unsure of your answer or do not understand the question, read the question again carefully and make a vertical mark ( **\|** ) on the line based upon your best understanding of the question. If you want to change your answer, please do the following: make a new vertical mark ( **\|** ); draw an arrow to the correct mark; initial and date the correction. | |
| Each question uses a visual analogue scale. Think about how you rate your feelings and place a vertical mark ( **\|** ) on the line at a point corresponding to what extent your nausea (or vomiting) has affected that aspect of your quality of life. **Please read the question carefully because in some questions, a “1” indicates no effect on your quality of life and in other questions a “1” indicates a great deal of an effect on your quality of life.** You may place your vertical mark ( **\|** ) at any point along the line. Be sure that you make your vertical mark ( **\|** ) so that it intersects the horizontal line. Do not circle a number. Use a single vertical mark ( **\|** ) as shown below. | |
| **Correct: Vertical mark** | |
| 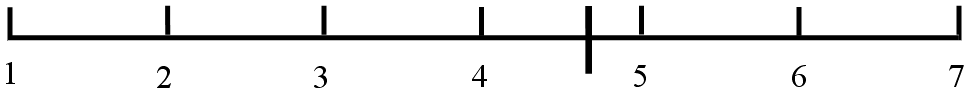 | |
| Not at all | A Great Deal |
| **Incorrect: "**🞬**"** | |
| 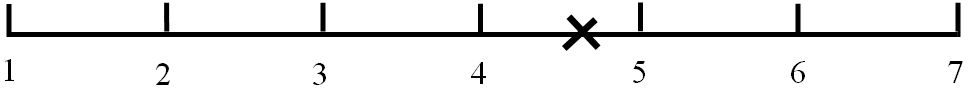 | |
| Not at all | A Great Deal |
| **Incorrect: Circle number** | |
| 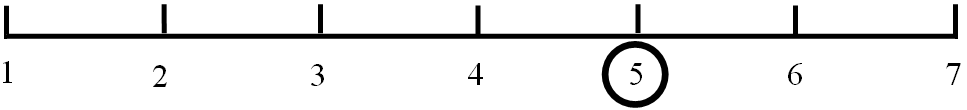 | |
| Not at all | A Great Deal |

Copyright © 1992 Celeste Lindley

All Rights Reserved

Functional Living Index Emesis

1. Have you had nausea in the past 5 days?

|  |  |  |  |  |  |
| --- | --- | --- | --- | --- | --- |

1 2 3 4 5 6 7

| Not at all | A Great Deal |
| --- | --- |

1. Has nausea affected your ability to continue your usual recreation or leisure activities in the past 5 days?

|  |  |  |  |  |  |
| --- | --- | --- | --- | --- | --- |

1 2 3 4 5 6 7

| Not at all | A Great Deal |
| --- | --- |

1. Has nausea affected your ability to prepare a meal or do minor household tasks in the past 5 days?

|  |  |  |  |  |  |
| --- | --- | --- | --- | --- | --- |

1 2 3 4 5 6 7

| A Great Deal | Not at all |
| --- | --- |

1. How much has nausea affected your ability to enjoy a meal in the past 5 days?

|  |  |  |  |  |  |
| --- | --- | --- | --- | --- | --- |

1 2 3 4 5 6 7

| Not at all | A Great Deal |
| --- | --- |

1. How much has nausea affected your ability to enjoy drinking fluids in the past 5 days?

|  |  |  |  |  |  |
| --- | --- | --- | --- | --- | --- |

1 2 3 4 5 6 7

| Not at all | A Great Deal |
| --- | --- |

1. How much has nausea affected your willingness to see and spend time with family and friends, in the past 5 days?

|  |  |  |  |  |  |
| --- | --- | --- | --- | --- | --- |

1 2 3 4 5 6 7

| A Great Deal | Not at all |
| --- | --- |

1. Has nausea affected your daily functioning in the past 5 days?

|  |  |  |  |  |  |
| --- | --- | --- | --- | --- | --- |

1 2 3 4 5 6 7

| Not at all | A Great Deal |
| --- | --- |

1. Rate the degree to which your nausea has imposed a hardship on you (personally) in the past 5 days.

|  |  |  |  |  |  |
| --- | --- | --- | --- | --- | --- |

1 2 3 4 5 6 7

| Not at all | A Great Deal |
| --- | --- |

1. Rate the degree to which your nausea has imposed a hardship on those closest to you in the past 5 days.

|  |  |  |  |  |  |
| --- | --- | --- | --- | --- | --- |

1 2 3 4 5 6 7

| Not at all | A Great Deal |
| --- | --- |

Functional Living Index Emesis

1. Have you vomited in the past 5 days?

|  |  |  |  |  |  |
| --- | --- | --- | --- | --- | --- |

1 2 3 4 5 6 7

| Not at all | A Great Deal |
| --- | --- |

1. Has vomiting affected your ability to continue your usual recreation or leisure activities in the past 5 days?

|  |  |  |  |  |  |
| --- | --- | --- | --- | --- | --- |

1 2 3 4 5 6 7

| A Great Deal | Not at all |
| --- | --- |

1. Has vomiting affected your ability to prepare a meal or do minor household tasks in the past 5 days?

|  |  |  |  |  |  |
| --- | --- | --- | --- | --- | --- |

1 2 3 4 5 6 7

| Not at all | A Great Deal |
| --- | --- |

1. How much has vomiting affected your ability to enjoy a meal in the past 5 days?

|  |  |  |  |  |  |
| --- | --- | --- | --- | --- | --- |

1 2 3 4 5 6 7

| Not at all | A Great Deal |
| --- | --- |

1. How much has vomiting affected your ability to enjoy drinking fluids in the past 5 days?

|  |  |  |  |  |  |
| --- | --- | --- | --- | --- | --- |

1 2 3 4 5 6 7

| Not at all | A Great Deal |
| --- | --- |

1. How much has vomiting affected your willingness to see and spend time with family and friends, in the past 5 days?

|  |  |  |  |  |  |
| --- | --- | --- | --- | --- | --- |

1 2 3 4 5 6 7

| A Great Deal | Not at all |
| --- | --- |

1. Has vomiting affected your daily functioning during the past 5 days?

|  |  |  |  |  |  |
| --- | --- | --- | --- | --- | --- |

1 2 3 4 5 6 7

| Not at all | A Great Deal |
| --- | --- |

1. Rate the degree to which your vomiting has imposed a hardship on you (personally) in the past 5 days.

|  |  |  |  |  |  |
| --- | --- | --- | --- | --- | --- |

1 2 3 4 5 6 7

| Not at all | A Great Deal |
| --- | --- |

1. Rate the degree to which your vomiting has imposed a hardship on those closest to you in the past 5 days.

|  |  |  |  |  |  |
| --- | --- | --- | --- | --- | --- |

1 2 3 4 5 6 7

| A Great Deal | Not at all |  |
| --- | --- | --- |

**PRO CTCAE and QoL EORTC QLQ-C15-PAL**

Study ID: ____________

Date of Completion: _________________

🞎 Baseline

🞎 2-week follow-up

🞎 4-week follow-up

**Please circle the answer that best describes your situation.**

Q1) In the last 7 days, how much did fatigue, tiredness, or lack of energy interfere with your usual or daily activities?

1 2 3 4 5 6 7

Not at all A Great Deal

Q2) In the last 7 days, what was the severity of your fatigue, tiredness, or lack of energy at its worst?

1 2 3 4 5 6 7

Not at all A Great Deal

Q3) In the last 7 days, what was the severity of your skin burns from radiation at their worst?

1 2 3 4 5 6 7

Not at all A Great Deal

Q4) How would you rate your overall quality of life during the past week?

1 2 3 4 5 6 7

Very Poor Excellent
