# Supplementary material for: SUPR-3D: A randomized phase iii trial comparing simple unplanned palliative radiotherapy versus 3d conformal radiotherapy for patients with bone metastases: study protocol
Source: BMC Cancer. 2019 Oct 28;19:1011. doi: 10.1186/s12885-019-6259-z (PMC6819327; doi:10.1186/s12885-019-6259-z)
Supplement: Supplementary file 1 — Additional file 1: Appendix 1. Eligibility criteria. Appendix 2. Patient reported outcomes. Appendix 3: HCP-reported baseline and follow-up. Appendix 4. Treatment related data. Appendix 5. Informed consent form. [file 12885_2019_6259_MOESM1_ESM.zip › APPENDIX CR3.docx]

**APPENDIX C – HCP-REPORTED BASELINE AND FOLLOW-UP (MEDICATIONS and TOXICITY)**

**Outcome data**

Study ID: ________________

Date of Assessment (Day, Month, Year): _______________________

🞎 Baseline

🞎 2-week follow-up

🞎 4-week follow-up

🞎 other

1. ECOG Status: 0 1 2 3 4 5 (please circle)
2. Medication use (within last 24 hours):

Include at least analgesics, bisphosphonates, anti-emetics, (e.g. 5HT3 antagonists such as Ondansetron) and steroids. No need to include patient’s non-cancer regular meds (e.g. antihypertensives).

| Name of medication | Dosage | Start date | End date |
| --- | --- | --- | --- |
|  |  |  |  |
|  |  |  |  |
|  |  |  |  |
|  |  |  |  |
|  |  |  |  |
|  |  |  |  |
|  |  |  |  |
|  |  |  |  |

1. Toxicity

**Toxicity (assess at baseline and at each FU appt) - CTCAE v5.0**

**Pain**

1. No Pain
2. Mild pain
3. Moderate pain; limiting instrumental ADL
4. Severe pain; limiting self-care ADL

**Fatigue**

1. No fatigue
2. Fatigue relieved by rest
3. Fatigue not relieved by rest; limiting instrumental ADL
4. Fatigue not relieved by rest; limiting self care ADL

**Diarrhea**

1. No diarrhea
2. Increase of <4 stools per day over baseline; mild increase in ostomy output
3. Increase of 4-6 stools per day over baseline, moderate increase in ostomy output
4. Increase of >=7 stools per day over baseline; incontinence; hospitalization indicated
5. Life-Threatening consequences; urgent intervention indicated
6. Death

**Nausea**

1. No nausea
2. Loss of appetite without alteration in eating habits
3. Oral intake decreased without significant weight loss, dehydration or malnutrition
4. Inadequate oral caloric or fluid intake; tube feeding, TPN or hospitalization indicated

**Investigator Name:** _______________________________________

**Investigator Signature:** ____________________________________

**Date:** ____________________________
