# Supplementary material for: SUPR-3D: A randomized phase iii trial comparing simple unplanned palliative radiotherapy versus 3d conformal radiotherapy for patients with bone metastases: study protocol
Source: BMC Cancer. 2019 Oct 28;19:1011. doi: 10.1186/s12885-019-6259-z (PMC6819327; doi:10.1186/s12885-019-6259-z)
Supplement: Supplementary file 1 — Additional file 1: Appendix 1. Eligibility criteria. Appendix 2. Patient reported outcomes. Appendix 3: HCP-reported baseline and follow-up. Appendix 4. Treatment related data. Appendix 5. Informed consent form. [file 12885_2019_6259_MOESM1_ESM.zip › APPENDIX D Treatment related dataR3.docx]

**APPENDIX D Treatment related data**

Study ID: ______________________

Planning

RO plan review performed: Y/N

Treatment

CBCT use: Y/N

Duration: CT sim – 1st RT:

(please circle)

< 1 day

< 2 days

< 3 days

< 4 days

< 5 days

> 5 days

**Investigator Name:** _______________________________________

**Investigator Signature:** ____________________________________

**Date:** ____________________________

**PROTOCOL SIGNATURE PAGE**

**Protocol Title:** SUPR-3D: A RANDOMIZED PHASE III TRIAL COMPARING SIMPLE UNPLANNED PALLIATIVE RADIOTHERAPY VERSUS 3D CONFORMAL RADIOTHERAPY FOR PATIENTS WITH BONE METASTASES

**Protocol Version/ Date**: _________________

**Sponsor/Study Principal Investigator Name:** Dr. Robert Olson

**Site**: _________________________________

**Declaration of Investigator**

I confirm that I have read the above-mentioned protocol and its attachments, and I agree to:

- conduct the described trial in compliance with all stipulations of the protocol and ICH E6 Guideline for Good Clinical Practice (GCP);
- comply with procedures for data entry/recording/reporting as outlined in the data management plan;
- permit monitoring, auditing and inspection; and
- retain the trial related essential documents until Dr. Robert Olson (Study Principal Investigator) informs me that these documents are no longer needed.

**Site Principal Investigator Name**: _________________________________________

**Site Principal Investigator Signature**: _____________________________________

**Date**: __________________________________________
