# Supplementary material for: SUPR-3D: A randomized phase iii trial comparing simple unplanned palliative radiotherapy versus 3d conformal radiotherapy for patients with bone metastases: study protocol
Source: BMC Cancer. 2019 Oct 28;19:1011. doi: 10.1186/s12885-019-6259-z (PMC6819327; doi:10.1186/s12885-019-6259-z)
Supplement: Supplementary file 1 — Additional file 1: Appendix 1. Eligibility criteria. Appendix 2. Patient reported outcomes. Appendix 3: HCP-reported baseline and follow-up. Appendix 4. Treatment related data. Appendix 5. Informed consent form. [file 12885_2019_6259_MOESM1_ESM.zip › APPENDIX E Informed Consent FormR3.docx]

**APPENDIX E Informed Consent Form**

**Participant Information and Consent Form**

**SUPR-3D: A RANDOMIZED PHASE III TRIAL COMPARING SIMPLE UNPLANNED PALLIATIVE RADIOTHERAPY VERSUS 3D CONFORMAL RADIOTHERAPY FOR PATIENTS WITH BONE METASTASES**

**Principal Investigators:** Dr. Robert Olson

BC Cancer – Centre for the North

250-645-7325

**Principal Investigators at Additional Participating BC Centres:**

**Fred Hsu, MD**  Radiation Oncology

BC Cancer Agency – Abbotsford Centre

Telephone Number: 604-851-4743

**Benjamin Mou, MD** Radiation Oncology

BC Cancer Agency – Kelowna

Telephone Number: 250-712-3911 Ext 686645

**Devin Schellenberg, MD** Radiation Oncology

BC Cancer – Surrey

Telephone Number: 604-930-4085

**Shilo Lefresne, MD** Radiation Oncology

BC Cancer - Vancouver

Telephone Number: 604-877-6000 Ext. 672673

**Tanya Berrang, MD** Radiation Oncology

BC Cancer Agency – Victoria

Telephone Number: 250-519-5426

**Joanna Laba, MD** Radiation Oncology

London Health Sciences Centre – London Ontario

Telephone Number: 519-685-8500

**Sponsors:** BC Cancer

**For emergencies only:** Call the centre nearest you and ask for your study doctor or, if he or she is not available, ask for your usual oncologist or the oncologist on-call.

Vancouver (604) 877-6000

Victoria (250) 370-8000

Surrey (604) 581-2211

Abbotsford (604) 851-4700

Kelowna (250) 862-4000

Prince George (250) 645-7300

**For non-emergency contact numbers:** See sections 19 and 20

1. **Invitation**

You are being invited to take part in this research study because your cancer has spread to the bone and you will receive radiotherapy (RT) as treatment for your symptoms related to the bone metastases.

1. **Your participation is voluntary**

Your participation is voluntary. You have the right to refuse to participate in this study. If you decide to participate, you may still choose to withdraw from the study at any time without any negative consequences to the medical care, education, or other services to which you are entitled or are presently receiving.

You should be aware that there is a difference for both you and your doctor between being a patient and being a research participant. As a patient all medical procedures and treatments are carried out for your benefit only according to standard accepted practice. As a research participant you and your doctor also must take into account the requirements for the research study. These may include procedures and treatments that are not part of standard practice or are not yet proven. This consent form describes the diagnostic and treatment procedures that are being carried out for research purposes. Please review the consent document carefully when deciding whether or not you wish to be part of the research and sign this consent only if you accept being a research participant.

If you wish to participate in this study, you will be asked to sign this form.

Please take time to read the following information carefully and to discuss it with your family, friends, and doctor before you decide.

1. **Who is conducting this study?**

This study is being conducted by the Department of Radiation Oncology at BC Cancer. This study is not receiving funds from an external agency or sponsor.

1. **Background**

Bone metastases are the most common site of distant metastases and can cause severe symptoms. These complications can greatly affect a patient’s quality of life and cause a significant amount of pain. Radiotherapy (RT) is an effective treatment for patients with painful bone metastases. SUPR (simple unplanned Palliative Radiotherapy) is currently the standard treatment technique for bone metastases in BC.

In radiotherapy, multiple techniques can be used. Examples of these are Simple Unplanned Palliative Radiotherapy (SUPR) and Volumetric Modulated Arc Therapy (VMAT). SUPR is the standard of care in BC. For the SUPR treatment technique, simple calculations are performed for the radiation plan and the radiation is delivered with simple radiation beams. The advantage of this, is a quick turn-over which means that patients can be treated (or start treatment) within 24 hours, while the downside is that nearby normal tissue is not spared from radiotherapy. Conversely, with VMAT, a form of 3-dimensional radiotherapy, more complex calculations are done in order to spare nearby normal tissue, and patients typically need to wait 1-3 days before treatment can be started.

This study will allow us to determine if there is reduced toxicity associated with VMAT compared to SUPR while also measuring the impact on resources.

This study will enroll 250 participants. Approximately 150 people will take part from BC Cancer.

1. **What is the purpose of the study?**

The primary purpose of this study is to compare patient reported quality of life related to nausea and vomiting (RINV) between standard palliative radiotherapy and VMAT and to further explore any benefits in terms of toxicity associated with VMAT compared to SUPR. Additionally, we will evaluate the rate of complete control of RINV, toxicity, and pain response.

1. **Who can participate in this study?**

You may be able to participate in this study if*:*

• You are 18 or older.

• You provide written informed consent.

• You have a diagnosis of cancer with bone metastases.

• You are going to receive palliative intent radiation therapy (RT).

• Your Radiation Oncologist believes it is safe to treat you with SUPR.

• You are able to do light activities.

• Your Radiation Oncologist believes you are healthy enough to participate in this study.

• You are able and willing to complete the questionnaires and other assessments that are a part of this study.

- You will be required to be on an anti-nausea medication for treatment.

1. **Who should not participate in this study?**

You will not be eligible to participate in this study if:

• You have other serious medical issues that could affect your ability to participate.

• There is evidence of spinal cord compression.

• You are a pregnant or breastfeeding.

• You require treatments outside standard clinical hours.

- You have had whole brain RT within 4 weeks of your bone metastases RT.
- You have an implanted electronic device within 10 cm of the RT fields.
- You have prostheses close to the radiation target area.
- You have had previous RT that requires an analysis of cumulative dosages.

1. **What does the study involve?**

If you agree to participate in the study, you will receive one of the two radiation treatments listed below. The process for assigning which treatment you will have is random (like flipping a coin) with equal chance of getting either treatment. Neither you nor your study doctor can choose which treatment you will get but it will be assigned in a random way by a computer.

1. Simple unplanned Palliative Radiotherapy (SUPR): This is standard of care palliative radiotherapy in BC. You require a CT scan to plan the radiotherapy, which usually takes 1 hour, and then usually you can receive radiotherapy within 24 hours. The treatment time is usually under 30 minutes.
2. Volumetric Modulated Arc Therapy (VMAT): Like standard of care SUPR, you will require a 1 hour CT scan to plan radiotherapy. The treatment is also usually on 30 minutes. The main difference you will notice is that the planning time would be up to 3 days,; the extra time required is one of the items we are studying.
3. **What are my responsibilities?**

If you agree to participate in the study, you will be expected to undergo the treatment assigned by the randomization process. We also expect that you will complete the questionnaires required for the study.

Participants unable to return to the clinic 5 days (if applicable), 2 weeks, and 4 weeks post treatment will have the option to complete questionnaires at home (online or on paper). Online questionnaires will be completed via PatientPortals.ca. BC Cancer is licensed by PatientPortals.ca to collect data. Patient registration and use of PatientPortals.ca is voluntary and free of charge for the patient.

1. **What are the possible harms and discomforts?**

In VMAT, the bone metastasis will be contoured (outlined) on the CT images and the radiation dose will be planned closely surrounding these contours. Therefore, there is a small theoretical risk of missing part of the metastasis, if this is not visible on CT or other imaging modalities. In SUPR, larger fields are being used, which results in a larger margin surrounding the metastasis. This reduces the risk of missing cancer cells.

We do not anticipate any additional harms or discomforts from participating in this study. There is a possibility due to increased planning time required there may be a slight delay in receiving treatment of 1 to 3 days. This delay in radiation may mean a delay in you receiving pain relief if you take part in this study.

1. **What are the potential benefits of participating?**

No one knows whether or not you will benefit from this study. There may or may not be direct benefits to you from taking part in this study.

We hope that the information learned from this study can be used in the future to benefit other people with a similar disease.

1. **What are the alternatives to the study treatment?**

If you choose not to participate in this study or to withdraw at a later date, the following treatment options may be available to you:

- SUPR (simple unplanned Palliative Radiotherapy) would still be made available to you. This is currently the standard treatment technique for bone metastases in BC.

You can discuss these options with your doctor before deciding whether or not to participate in this research project.

1. **What if new information becomes available that may affect my decision to participate?**

If you choose to enter this study and at a later date a more effective treatment becomes available, it will be discussed with you. You will also be advised of any new information that becomes available that may affect your willingness to remain in this study.

1. **What happens if I decide to withdraw my consent to participate?**

You may withdraw from this study at any time without giving reasons. If you choose to enter the study and then decide to withdraw at a later time, you have the right to request the withdrawal of your information collected during the study. This request will be respected to the extent possible. Please note however that there may be exceptions where the data will not be able to be withdrawn for example where the data is no longer identifiable (meaning it cannot be linked in any way back to your identity) or where the data has been merged with other data If you would like to request the withdrawal of your data, please let your study doctor know.

1. **Can I be asked to leave the study?**

If you are not able to follow the requirements of the study or for any other reason, the study doctor may withdraw you from the study and will arrange for your care to continue. On receiving new information about the treatment, your research doctor might consider it to be in your best interests to withdraw you from the study without your consent if they judge that it would be better for your health. If you are asked to leave the study, the reasons for this will be explained to you and you will have the opportunity to ask questions about this decision.

1. **How will my taking part in this study be kept confidential?**

Your confidentiality will be respected. However, research records and health or other source records identifying you may be inspected in the presence of the Investigator by representatives of BC Cancer and the BC Cancer Research Ethics Board for the purpose of monitoring the research. No information or records that disclose your identity will be published without your consent, nor will any information or records that disclose your identity be removed or released without your consent unless required by law.

You will be assigned a unique study number as a participant in this study. This number will not include any personal information that could identify you (e.g., it will not include your Personal Health Number, SIN, or your initials, etc.). Only this number will be used on any research-related information collected about you during the course of this study, so that your identity will be kept confidential. Information that contains your identity will remain only with the Principal Investigator and/or designate. The list that matches your name to the unique study number that is used on your research-related information will not be removed or released without your consent unless required by law.

Your rights to privacy are legally protected by federal and provincial laws that require safeguards to insure that your privacy is respected. You also have the legal right of access to the information about you that has been provided to the sponsor and, if need be, an opportunity to correct any errors in this information. Further details about these laws are available on request to your study doctor.

Your family physician will be notified of your participation in the study so that your study doctor and your family doctor can provide proper medical care.

# **Registration of Clinical Trials**

A description of this clinical trial will be available on [www.clinicaltrials.gov](file:///\\joker\trials\breast\ma.39\Consent\www.clinicaltrials.gov). This website will not include information that can identify you. You can search this website at any time.

1. **What happens if something goes wrong?**

By signing this form, you do not give up any of your legal rights and you do not release the study doctor, participating institutions, or anyone else from their legal and professional duties. If you become ill or physically injured as a result of participation in this study, medical treatment will be provided at no additional cost to you. The costs of your medical treatment will be paid by your provincial medical plan.

1. **What will the study cost me?**

All research-related medical care and treatment and any related tests that you will receive during your participation in this study will be provided at no cost to you.

**Reimbursement**

You will not be reimbursed for study-related expenses such as specify, e.g., parking, etc.

**Remuneration**

You will not be paid for participating in this study.

1. **Who do I contact if I have questions about the study during my participation?**

If you have questions about taking part in this study or if you suffer a research-related injury you can talk to your study doctor, or family doctor. If you suffer a study-related injury you should immediately talk to your study doctor, or if he or she is not available the oncologist on call. Your study doctor is:

|  |  |  |
| --- | --- | --- |
| Name |  | Telephone |

Or, you can speak to the doctor who is the BC Cancer principal investigator, Dr. Rob Olson, at 250-645-7325.

Or, you can speak to the Head of the Radiation Therapy Program of BC Cancer at 604-877-6000.

1. **Who do I contact if I have any questions or concerns about my rights as a participant?**

If you have any concerns or complaints about your rights as a research participant and/or your experiences while participating in this study, contact the Research Participant Complaint Line in the University of British Columbia Office of Research Ethics by e-mail at [RSIL@ors.ubc.ca](mailto:RSIL@ors.ubc.ca) or by phone at 604-822-8598 (Toll Free: 1-877-822-8598). Please reference the study number H18-01938 when contacting the Complaint Line so the staff can better assist you

1. **Signatures**

**SUPR-3D: A RANDOMIZED PHASE III TRIAL COMPARING SIMPLE UNPLANNED PALLIATIVE RADIOTHERAPY VERSUS 3D CONFORMAL RADIOTHERAPY FOR PATIENTS WITH BONE METASTASES**

**Participant Consent**

My signature on this consent form means:

- I have read and understood the information in this consent form.
- I have had enough time to think about the information provided.
- I have been able to ask for advice if needed.
- I have been able to ask questions and have had satisfactory responses to my questions.
- I understand that all of the information collected will be kept confidential and that the results will only be used for scientific purposes.
- I understand that my participation in this study is voluntary.
- I understand that I am completely free at any time to refuse to participate or to withdraw from this study at any time, and that this will not change the quality of care that I receive.
- I authorize access to my health records as described in this consent form.
- I am providing my email address and would like to be setup to complete the study questionnaires online via PatientPortals.ca: Email address: _______________________________
- I understand that I am not waiving any of my legal rights as a result of signing this consent form.
- I understand that there is no guarantee that this study will provide any benefits to me.

I will receive a signed copy of this consent form for my own records.

I consent to participate in this study.

_____________________________________________________________________________

Participant’s Signature Printed Name Date

_____________________________________________________________________________

Signature of Person Printed Name Study Role Date

Obtaining Consent

If this consent process has been done in a language other than that on this written form, with the assistance of an interpreter/translator, indicate:

Language: ____________________

Was the participant assisted during the consent process in one of ways listed below?

□ Yes □ No [Note: For typical situations where the person conducting the consent discussion simply reads the consent with the participant to ensure that informed consent is properly obtained, check “no”.]

If yes, please check the relevant box and complete the signature space below:

□ The consent form was read to the participant, and the person signing below attests that the study was accurately explained to, and apparently understood by, the participant (please check if participant is unable to read).

□ The person signing below acted as an interpreter/translator for the participant, during the consent process (please check if an interpreter/translator assisted during the consent process).

___________________________ ________________________ _______________

Signature of Person Assisting Printed Name Date

in the Consent Discussion

# APPENDIX F: World Health Organization Trial Registration Dataset

| **Item** | **Description** |
| --- | --- |
| Primary registry and trial identifying number | ClinicalTrials.gov NCT03694015 |
| Date of registration in primary registry | 3 October, 2018 |
| Secondary identifying numbers | N/A |
| Source(s) of monetary or material support | BC Cancer Foundation & BC Cancer Agency |
| Primary sponsor | British Columbia Cancer Agency |
| Secondary sponsor(s) | N/A |
| Contact for public queries | Robert Olson MD FRCPC Msc, rolson2@bccancer.bc.ca |
| Contact for scientific queries | Robert Olson MD FRCPC Msc  BC Cancer – Prince George, BC, Canada |
| Public title | SUPR-3D: Simple Unplanned Palliative Radiotherapy Versus 3D Conformal Radiotherapy for Patients With Bone Metastases |
| Scientific title | SUPR-3D: A Randomized Phase III Trial Comparing Simple Unplanned Palliative Radiotherapy Versus 3D Conformal Radiotherapy for Patients With Bone Metastases |
| Countries of recruitment | Canada |
| Health condition(s) or problem(s) studied | Neoplasm metastasis, neoplastic processes, neoplasms, pathologic processes |
| Intervention(s) | SUPR  simple unplanned palliative radiotherapy-(either 8 Gy in 1 fraction or 20 Gy in 5 fractions), chosen pre-randomization at ROs or centres discretion |
|  | VMAT  volumetric modulated arc therapy--(either 8 Gy in 1 fraction or 20 Gy in 5 fractions), chosen pre-randomization at ROs or centres discretion |
| Key inclusion and exclusion criteria | Inclusion Criteria:  Able to provide informed consent; Clinical Diagnosis of cancer with bone metastases (biopsy not required); Currently being managed with palliative intent RT to 1-3 bone metastases, at least one of which must (at least) partly lie within T11-L5 or pelvis; ECOG Performance Status 0-3; Radiation Oncologist is comfortable prescribing 8 Gy in 1 fraction or 20 Gy in 5 fractions RT for bone metastases; Pregnancy test for women of child-bearing age;  Patient is able (i.e. sufficiently fluent) and willing to complete the quality of life questionnaire in either English or French. The baseline assessment must be completed within required timelines, prior to randomization; Patients must be accessible for treatment and follow-up. Investigators must assure themselves the patients randomized on this trial will be available for complete documentation of the treatment, adverse events, and follow-up.  Patient must fulfill treatment specific eligibility criteria . Plans not meeting the treatment specific eligibility requirements are not eligible for VMAT Rapid; No previous treatments requiring EQD2/sum plan constraints; No pacemaker within 10 cm of the RT fields; No contrast required; No mould room necessary (except shells); No significant hardware within the treatment field  maximum of 2 OARs requiring optimization; For VMAT: PTV volume > 35 cm^3 (2.0 cm radius sphere, roughly 4x4 field)  For SUPR: at least 4x4 field |
|  | Exclusion criteria:  Bone metastases being treated is not (at least) partly located in T11 - L5 or pelvis; Patients whom radiation oncologist is prescribing a dose other than 8 Gy in a single fraction or 20 Gy in 5 fractions; Serious medical co-morbidities precluding radiotherapy  Clinical evidence of spinal cord compression; Spinal cord in treatment field has already received at least >30 Gy EQD2; Solitary plasmocytoma; Pregnant or lactating women; Target volume cannot be encompassed by a single VMAT isocentre; No custom bolus; Patients requiring treatments outside standard clinical hours |
| Study type | Interventional Allocation: Randomized Intervention model description: Simple randomization with stratification will be used to randomly assign patients to either Arm 1 or Arm 2 in a 1:1 ratio using a computer-generated randomization scheme. Masking: None (Open Label) Primary purpose: Treatment |
| Date of first enrolment | July 2019 |
| Target sample size | 250 |
| Recruitment status | Not yet recruiting |
| Primary outcome(s) | Patient Reported Quality of Life related to Radiation Induced Nausea and Vomiting as measured by Functional Living Index - Emesis |
| Key secondary outcomes | Control of Radiation Induced Nausea and Vomiting  Pain Flare  Medication use  PRO-CTCAE |
